# Adoption of new health products in low and middle income settings: how product development partnerships can support country decision making

**DOI:** 10.1186/1478-4505-9-15

**Published:** 2011-03-31

**Authors:** William A Wells, Alan Brooks

**Affiliations:** 1 Global Alliance for TB Drug Development, 40 Wall St, New York NY 10005 USA; 2 Previous Address: PATH Malaria Vaccine Initiative, Bâtiment Avant Centre, 13 chemin du Levant 01210, Ferney-Voltaire, France; 3 Current Address: Swiss Tropical and Public Health Institute, Socinstrasse 57, 4051 Basel, Switzerland

## Abstract

When a new health product becomes available, countries have a choice to adopt the product into their national health systems or to pursue an alternate strategy to address the public health problem. Here, we describe the role for product development partnerships (PDPs) in supporting this decision-making process. PDPs are focused on developing new products to respond to health problems prevalent in low and middle income settings. The impact of these products within public sector health systems can only be realized after a country policy process. PDPs may be the organizations most familiar with the evidence which assists decision making, and this generally translates into involvement in international policy development, but PDPs have limited reach into endemic countries. In a few individual countries, there may be more extensive involvement in tracking adoption activities and generating local evidence. This local PDP involvement begins with geographical prioritization based on disease burden, relationships established during clinical trials, PDP in-country resources, and other factors. Strategies adopted by PDPs to establish a presence in endemic countries vary from the opening of country offices to engagement of part-time consultants or with long-term or ad hoc committees. Once a PDP commits to support country decision making, the approaches vary, but include country consultations, regional meetings, formation of regional, product-specific committees, support of in-country advocates, development of decision-making frameworks, provision of technical assistance to aid therapeutic or diagnostic guideline revision, and conduct of stakeholder and Phase 4 studies. To reach large numbers of countries, the formation of partnerships, particularly with WHO, are essential. At this early stage, impact data are limited. But available evidence suggests PDPs can and do play an important catalytic role in their support of country decision making in a number of target countries.

## Introduction

For health innovations to have their full impact, they must reach those in need. This job of achieving access is a multifaceted endeavor [1-3] requiring consideration of issues such as financing [4], manufacturing [5], pricing [6], international policies [7], regulatory approval [8], translational research [9], end-user acceptance [10] and a strategic communication approach to decision makers [11]. Here we consider country decision making - the process by which a country weighs evidence and decides whether or not to adopt a new product into its national guidelines and practice [12,13]. Such policy decisions are necessary, but not sufficient, for subsequent implementation and public health impact [14], as has been shown by the delays from decision to implementation of artemisinin-based combination therapies for malaria treatment [15].

Although the policy change process is ultimately controlled by the country, in low and middle income settings in particular there are many other actors who provide input and can strengthen local decision making [16]. One such set of actors is the product development partnerships (PDPs). These not-for-profit organizations were formed because commercial incentives had proven insufficient to draw for-profit companies into certain important areas, such as drug development for tuberculosis (TB), malaria, sleeping sickness and visceral leishmaniasis, vaccine development for HIV, TB, malaria, dengue fever, meningococcal meningitis and pneumonia, microbicide development for HIV, and insecticide development for vector-borne diseases [17].

Over the last several years, these not-for-profit organizations have catalyzed the production of an increasing number of health products designed specifically for use in low and middle income settings, such as drugs for malaria and trypanosomiasis and diagnostics for TB. Although PDPs have been focused primarily on product development, they share a vision of realizing the public health impact promised by new products. PDPs have generally translated this vision into catalytic support both for global policy change and, in a limited number of countries, for activities which could lead to decisions on use, and therefore support uptake and introduction of the resulting products. Such activities are implemented in partnership with endemic countries and other organizations.

Here, we explore how the PDPs can assist in the country decision making aspect of product introduction. Although we recognize the leadership of other actors including, most notably, country stakeholders themselves, our primary focus for this article is on the role of PDPs. Certain other organizations, such as the Hib Initiative, have focused on product introduction specifically, rather than on product development; these are considered more briefly for relevant lessons. After defining country decision making, we investigate the various roles for PDPs, how those roles are prioritized geographically, the partnerships required, and the specific approaches used by a number of PDPs. This paper provides a set of baseline insights into support by PDPs for country decision-making. It will not define exactly what should be done in each situation, but provides extensive, concrete examples of what has been done and analyses why these approaches were chosen. It also summarizes the initial, as yet limited, data evaluating the impact of such work by PDPs. Although the role of PDPs in supporting country decision making is still evolving, we find some themes that we believe will be generally applicable for future efforts.

## How PDPs fit into country decision making

### Core and variable contributions by PDPs

A definition of country decision making will be a useful starting point, before discussing a possible PDP role. Previously, country decision-making on new health interventions has been investigated from a variety of perspectives. This has resulted in decision-making frameworks [14,18-21], guidance on introduction [3,22], and descriptions of the types of evidence considered [12,23,24] or the methods [25] or processes [26] used for considering them. Multi-variate analyses have suggested which interventions are related to faster uptake [27]. Although some authors have described supportive roles played by outside actors [16,20,21], in general the possible mechanisms for PDPs to support country decision making remain largely undocumented.

Decision-making processes occur within the unique socio-political and economic context of an individual country, whereas a multi-country analysis such as this one must rely instead on broad process categories (see below). The relative importance of other elements influencing decision-making, such as media pressure, corruption and politics, may vary greatly from country to country [28], and therefore are not addressed in detail here. Furthermore, we acknowledge that in any political system the use of evidence-based decision making is a goal, and not always a reality.

For the purposes of this paper, we have adopted a simplified framework, assuming country decision making requires background information (to elicit problem identification [29]), evidence to feed into decision making, and a process to consider that evidence. Within the information and evidence categories are many sub-categories to be considered by country stakeholders, including public health priority, disease burden, efficacy, quality, safety, comparison with other available interventions, presentation, supply (procurement and distribution), financial impact, and programmatic strength [22]. The process category includes, critically, the identification and convening of a sufficiently broad group of stakeholders who have the mandate and resources to identify and review evidence; this group can be under the aegis of the government (a specific disease control program, the broader Ministry of Health, the Ministry of Finance, a specific agency for technical assessment, or some combination of these agencies) or independent of but mandated by the government [13,16]. The final result should be a policy conclusion and, if the decision is supportive, implementation. At the center of this process is the country itself, supported by a range of local stakeholders and partner organizations.

As noted by Frost and Reich, "the production of acceptance [is] an active process of social construction, not a passive process of waiting for various experts to agree on key elements related to the use of a health technology" [1]. If this active process is missing or under-resourced, effective country decision making is jeopardized. Indeed, many challenges have arisen during country decision making. For example, malaria regimen change in Kenya was delayed due to a lack of national and international standards (e.g., varied criteria for chloroquine sensitivity and efficacy markers), insufficient evidence (of side-effect incidence and the true costs of new drug implementation), no national compendium of relevant data, uncoordinated local research studies that could not be compared to each other, and ineffective mechanisms for communicating research evidence to sub-national stakeholders. This resulted in national guideline change happening 6 years after a call for evidence review and 14 years after the first local data on resistance [30], with the consequence that millions of patients were treated with ineffective regimens. In the area of TB regimens, the current data available (e.g., on drug resistance patterns) are useful for programmatic monitoring of current TB treatment but leave gaps for decision-making around future regimen changes [31]. On the process side, there were delays in another malaria regimen change due to insufficiently broad participation in decision making (e.g., exclusion of local manufacturers, the Ministry of Finance, and sub-national implementers) [32]. Similarly, the change from 8- to 6-month TB regimens was delayed in high burden countries not only by insufficient evidence but also, in some countries, by unclear or non-existent procedures or bodies to consider regimen change [12].

Table 1 presents examples of how PDPs can contribute to the core activities of country decision making. These contributions build on the familiarity of PDPs with the research programs and evidence base surrounding a particular intervention.

**Table 1:** Examples of country decision making activities

Country activity	PDP support role
Background information	

• Define national health priorities.	• Conduct research to understand priority of disease area, generally, and likely desire for proposed product, specifically.
• Obtain information on the future products that are likely to become available.	• Disseminate product and pipeline information.

Process and people	

• Ensure a decision-making body (or person) is identified, active, and has members empowered to make decisions based upon available evidence.	• Facilitate awareness raising and transparent information sharing among appropriate stakeholders.
• Define a clear, step-wise and timely process for country decision-making in general (in a particular disease or intervention area) and then for adoption of new products specifically.	

Evidence base	

• Define the specific evidence base required for decision making, including local data requirements.	• Determine what information is expected to be needed for national decision-making (e.g., what efficacy endpoints).
• Make plans to generate this required local evidence base.	• Integrate consideration of these information needs into R&D activities. This affects, for example, clinical trial planning, development of regulatory strategies, and post-introduction strategies to monitor safety and impact.
	• Assist countries to define data needs and gaps, including clarifying if the information (e.g., on program and budget impact) should be generated in a country or internationally.
	• Gather and disseminate a standard evidence package informing decision making, or see that others do so. The data should come from a source or partnership that is credible to countries.
	• Train key personnel to respond to questions about the data or lack thereof.
	• Address concerns that are common across countries (e.g., price, cost-effectiveness, ease of use, source and geography of manufacturing, and impact on supply chain and existing program delivery).

There are a variety of additional PDP activities that can support country decision making but are only important under certain circumstances (Table 2). Such PDP access strategies and activities are project specific and focus on identified gaps: an awareness gap requires burden of disease studies or advocacy; whereas an evidence gap may require a cost-effectiveness study or operations research. Some of these activities require substantially more investment by a PDP. Furthermore, it cannot be assumed that global activities will reach policy makers in each country.

**Table 2:** Examples of additional support activities by PDPs

Additional support activity by PDP	Situation when needed or not needed
Background information	

Investigate disease burden, and share information with policy-makers.	Less need if disease is well characterized and recognized, and if there is already sufficient baseline surveillance to monitor impact.

Prioritize product introduction activities geographically based on disease burden, resistance patterns, or risk in specific populations.	Less relevant if disease is widespread and resistance patterns and risk factors vary little.

Process and people	

Catalyze the establishment of decision-making structures.	More need if bridging two fields in public health (e.g., immunization and malaria); less need if strong, defined structures already exist.

Support local advocacy or communications activities to inform policy makers about a disease and/or options for addressing a disease.	Depends on involvement of others who may undertake this, e.g., WHO and/or global disease partnerships. Global communications cannot be assumed to reach country level.

Evidence base	

Influence key aspects of the product development process that impact decision-making, such as pricing, supply, financing and regulatory issues, and demand estimation. Support the development and sharing of international policies, and of a post-introduction surveillance plan.	Depends on specific role of PDP in a country and in developing the product.

Generate or compile local evidence required for decision making, potentially including the funding and/or running of Phase 4 studies or operations research.	Depends on clearly defined needs from a country, whether the country can act as a regional or global source of data, and the willingness, local staffing, and available resources from PDP.

Support countries to make decisions about a complementary mix of interventions.	Only relevant if other interventions for the disease are widely used or being considered.

A number of these PDP activities are focused on capacity building for decision making in general, including the capacity for a country to decide not to adopt a PDP-related intervention. For example, a PDP can identify current gaps and highlight future decision-making needs, thus helping to catalyze the establishment of decision-making structures or processes [33]. These capacity building efforts are likely to be insufficient for health systems in general, however, given the limited geographic and product foci of PDPs, and the lack of mandate from the PDPs' donors for open-ended capacity building. For example, the categories in Tables 1 and 2 and the case studies provided below are based on the PDPs' analyses of the bottlenecks within a specific modality and disease area. Thus, the activity of other organizations to implement broader capacity building efforts, such as the establishment of National Immunization Technical Advisory Groups (NITAGs) [34], will remain critical and can be complemented by the work of PDPs.

Experience from the Hib Initiative suggests that generating data, and bringing the data to the attention of country stakeholders, may be an important part of catalyzing decision-making [11]. PDPs are one contributor to this activity, as part of the complex decision-making environment described previously. Each of the many stakeholders will bring some perspective, history, and perceived conflict. For PDPs, they may be seen as contributing a product or technology-biased emphasis. In contrast, a local researcher may emphasize the need for additional studies for which he or she would be funded, or a government official may be under pressure from a politician. It is critical to note that PDPs, to the authors' knowledge, do not have direct profit motives, unlike manufacturers, when supporting decision-making. The underlying rationale for PDPs is to help address a public health problem, for which the intervention arising with PDP support can be evaluated by a country for its role, or not, as one locally appropriate solution.

### Basis in research

Country decision-making, if it is evidence based, should be a mechanism to link research to policy. For PDPs, such a link forms most readily in countries where PDP-sponsored clinical trials are underway. In turn, a PDP's access strategy, and in particular its approach to country decision making, often emerges from engagement at country level during the clinical trial stage.

During the clinical trial approval process, PDPs naturally form links with countries via national regulatory authorities and ethics committees. But it is local researchers who generally lead the local implementation of PDP-supported clinical trials. To date, PDPs have found that it is important to proactively support a link between these local researchers and other endemic country stakeholders. This link can be through formal committees from the conception of the project and during the clinical trial phase, or via regular, informal briefings. If initiated early enough, this allows the PDP to share relevant background information with the country, increase the trust in and interpretability of resulting trial data, and ensure the product developed meets the country's needs. Local stakeholders can become partners in the project and can help to build country ownership of, and familiarity with, a product.

Once it is time for a decision, local researchers (rather than the PDPs) are best suited to present evidence to local decision makers and communicate directly with a government agency. Furthermore, it is more credible for the PDP to engage local stakeholders on technical grounds, and to provide them with the technical arguments they need so that they (rather than the PDP) can take part in the later, more political parts of the decision making process. In countries where no clinical trials are underway, the scientific and academic community is a key partner and translator of research findings in policy discussions.

### More extensive PDP involvement: The access architect and local evidence

Ultimately, it is country stakeholders who drive the two critical processes: defining what evidence base is necessary for product adoption and launch; and making the decision itself. The extent to which international actors assist this process depends on the country: it occurs more in the lowest income countries and less in more technically experienced, research-intensive countries [12]. At the country level, as at the global level, the PDP can provide information on programmatic implications and a standardized public health case weighing the evidence for and against adoption. This may represent a time-limited, targeted commitment from a PDP.

Typically, however, passive provision of this information is not enough to lead to clear country decisions [1]. Financing solutions are a key additional requirement, and there is often a need for an organization specifically responsible for tracking and coordinating all of the activities needed for access, including those activities needed before decision makers will reach an adoption decision. Others have referred to such an organization as providing the "architecture" for access [1]. PDPs may either be prominent in this role or it may be taken by others such as a local research institute, an implementing NGO, or an international partner such as the World Health Organization (WHO) (see section below on partnership).

Whichever organization is primarily responsible for supporting decision-making, they must interact extensively and directly with country stakeholders, particularly in countries identified as potential early adopters. This is not a role to be undertaken lightly.

The other, often overlapping area in which PDPs may become more heavily involved is the generation of local evidence. PDPs have an awareness of the evidence currently available, so they can help countries to determine which data need to be generated at a local level, versus provided from global studies. To support local processes, PDPs can also introduce models that can be adapted to generate local data for multiple countries (e.g., the International Vector Control Consortium (IVCC)'s monitoring tool (see below) and the PATH Malaria Vaccine Initiative (MVI)'s impact and cost models). Finally, PDPs can minimize the need to generate local data by uncovering existing local data at universities (programs and WHO may be unaware of these data) and by explaining why certain data were not needed if they would not affect the final decision.

Some PDPs devote considerable resources to the generation of local data; others devote almost none. This variability is determined in part by the earlier gap analysis - local data may or may not exist or be needed. The effort to generate local data is also determined by how novel an intervention is, either within its disease arena (e.g., a malaria vaccine coming into malaria control raises a number of local data questions) or by the existence of a program with clear accountability in the country which is already collecting such data (e.g., TB programs already hold extensive data on existing treatment regimens).

When PDPs do engage in these local processes, it is important that they do so with a health system rather than single product perspective. The PDP should define how the new intervention will fit with, affect, and strengthen other aspects of the existing public health environment, including all current and potential strategies to address a disease. This is how decision-makers in disease control programs think and they will be more likely to engage with the PDP if such an approach is demonstrated [35]. A disease approach can also bring in allies from other areas that might otherwise be competitors. An example of this is the Introducing New Approaches and Tools (INAT) sub-working group of the Stop TB Partnership, which looks at ways to encourage the adoption both of new technologies and of new guidelines and practices [36]. Finally, if necessary, PDPs and other partners should facilitate the formation of a normative decision making process that covers all interventions in an area, not just those sponsored by the PDP [16].

## The geography of PDP support of country decision making

### Role of international processes

International institutions reach decisions and issue guidelines that are often precursors to country-level decision making. For example, WHO recommendation will be essential for adoption of PDP-related products in many if not all of the relevant endemic countries. PDPs generally interact extensively with WHO to discuss what evidence is available or needs to be generated for the international guidelines process.

For some new health interventions, there is no specific, historical pathway for establishing international policies. This was the case for a candidate malaria vaccine, so MVI analyzed past WHO policy processes for vaccines and malaria interventions, identified data that could be required for a policy on malaria vaccines, and considered additional options for adjusting policy processes to accommodate a malaria vaccine [7]. PDPs can support the formation of international advisory and advocacy boards such as the Pneumococcal Awareness Council of Experts (PACE) and the All-Party Parliamentary Group on Pneumococcal Disease Prevention in the UK. These initiatives were instrumental in supporting the GAVI Alliance investment case and advance market commitment (AMC). It is reasonable to assume that working with or modifying existing policy processes, where feasible, will be less costly and more efficient for international partners than developing new processes.

### How many countries can a PDP reach?

The number of individual countries with which a PDP can expect to interact directly is not clear. It seems likely that most PDPs would interact significantly with perhaps 5-8 countries - notably those that have high disease burdens, are potential early adopters and are countries where the PDP is supporting clinical trials - but many PDPs would then rely on existing multilateral, NGO, and pharmaceutical partners to reach other countries for detailed work on adoption and implementation.

At one extreme is the PDP-like Hib Initiative, which worked together with WHO to support directly or indirectly 72 countries [11]. GAVI supported this group with a four-year, $37 million grant, after noting the existing 15-20 year uptake delay in most low income countries. The Hib Initiative had no real role in product development but was funded to support evidence-driven decisions on *Haemophilus influenzae *type b (Hib) vaccine use at global, regional and country levels. Their approach, which involved engaging directly or indirectly with a larger number of countries, was seen to be necessary to implement a global recommendation for use of a long-available product. Substantial savings may be realized and less engagement needed if appropriate steps - on financing, global policy and market preparation, for example - are taken during product development.

### Prioritizing countries for access engagement

If a PDP is going to invest significantly in supporting a country, it first needs to determine which countries are the highest priority. Prioritization generally considers two goals, which may or may not overlap: maximizing final public health impact; and identifying early adopters. Prioritization may also reflect an effort to include settings reflecting the full range of epidemiologic patterns of the disease (e.g., for malaria).

Prioritizing countries for engagement on access-related issues is not a science - there is no perfect answer. Different PDPs are likely to use shorter or longer lists of criteria in making such decisions. However, most PDPs will consider a number of the following criteria, with the more important listed first: prior engagement via PDP-sponsored trials; high burden of disease (absolute or reflecting specific patterns of resistance or vulnerability); potential health benefit (e.g., based on drug resistance patterns); political stability; capacity of national program to deliver treatment (e.g., focusing new vaccine interventions on those countries with strong EPI programs); existence of local champions and openness to change; research capability for a pilot, which would generate evidence for other countries; regional importance of country; regulatory capacity and influence; and availability of other information for decision making.

WHO regional advisers can also provide prioritization guidance. In support of the introduction of Hib vaccine, WHO regional EPI officers helped the Hib Initiative to identify issues and barriers for each country and define whether country stakeholders were already including Hib in their multi-year plans. A country's application to the Global Fund to Fight AIDS, TB and Malaria can provide similar insights. History can provide some hints about likely future actions [12], although the history of Hep B adoption provided the Pneumococcal Vaccines Accelerated Development and Introduction Plan (Pneumo-ADIP [9]) with few clues for the introduction of the pneumococcal vaccines, probably because of turnover of decision makers and different local champions for the two vaccines.

Once countries are prioritized, they may express an interest in conducting demonstration projects. As product developers, PDPs are focused on generating evidence from randomized, controlled clinical trials. Often, however, such evidence is not sufficient for adoption. After Phase 3 trials are complete, a deep investment may be needed in a few countries to help generate examples or evidence that an intervention works under real-world conditions (e.g., community effectiveness). Although countries vary, such test cases can boost the profile of a new intervention and result in funding for roll out in further countries. PDPs may help to build these early success cases and formulate roll-out plans.

### Strategies for establishing a local presence

Once a country has been prioritized, a PDP must decide whether and how to establish a local presence. A PDP with staff in an endemic country will have clear opportunities for improved information flow and closer engagement with local stakeholders. However, PDPs were primarily founded as research organizations, so budgets for an endemic country presence are usually driven by organizational needs such as those related to clinical trials. Aside from the Medicines for Malaria Venture (MMV)'s office in Uganda, offices associated with PDPs are not generally set up primarily for an access-related purpose.

The options for establishing a local presence, listed from most to least committed, include: country or regional offices, including with a partner organization; consultants on partial retainer; sustained engagement with existing committees or structures; engagement with ad hoc committees or structures formed at the prompting of a PDP; or ad hoc engagement with existing structures (e.g., regional or sub-regional disease-specific committees or meetings organized by WHO).

Local expertise, in the form of either PDP staff or consultants, is critical to ensure a high quality interaction with government officials and partner organizations. For example, disease-specific and health systems knowledge, and sufficient standing to collaborate with government officials, physicians and researchers are important. Individuals with such skills can help to design and facilitate local research and to provide local researchers and policy makers with PDP support for decision-making activities. Even beyond their own staff and consultants, PDPs may also help to strengthen the pool of scientists, who at the same time become stronger advocates within their regions and countries; examples include MVI's annual Malaria Vaccine Advocacy Fellowship Program, the Drugs for Neglected Diseases Initiative (DNDi)'s research platforms, or Pneumo-ADIP and Hib Initiative's local advocates.

PDPs vary in their in-country presences (Table 3), with the extent usually increasing as products move further through the pipeline. PDPs within larger institutions such as PATH are also able to leverage competencies in multiple offices.

**Table 3:** PDP offices in endemic countries

PDP	Offices in endemic countries
Drugs for Neglected Disease Initiative (DNDi)	Kenya, Brazil, Democratic Republic of the Congo, India, Malaysia, where it may be as small as a 1-person office

PATH Malaria Vaccine Initiative (MVI)	PATH office in Kenya with dedicated MVI program staff, plus PATH offices that can be called upon such as in Senegal, Ghana, Ethiopia, Uganda, Tanzania, Zambia, and South Africa

Medicines for Malaria Venture (MMV)	Uganda, initially for pilot of AMFm (Affordable Medicines Facility - malaria), now for regional interactions on guideline revisions

Institute for One World Health (iOWH)	India

Global Alliance for TB Drug Development (TB Alliance)	South Africa - focused on clinical trial conduct rather than access

Foundation for Innovative New Diagnostics (FIND)	India, Uganda

International AIDS Vaccine Initiative (IAVI)	India, Kenya, South Africa

Aeras Global TB Vaccine Foundation (Aeras)	South Africa

International Partnership for Microbicides (IPM)	South Africa

In sum, the rationale for the country presence of PDPs has been driven by research and development (R&D) or organizational needs (including limited funding), with access activities building upon that presence. This suggests that access teams should seek to be part of decisions on where R&D activities are undertaken. PDPs have also tended to take advantage of less costly means for engaging with greater numbers of countries by working through existing committees and structures (including WHO), developing new structures for sustained engagement where feasible, or otherwise working through ad hoc collaborations.

## The essential role of partnership

For PDPs to achieve their goals, partnership is essential. PDP access staff can initiate these partnerships by bringing together different parties, such as scientists, manufacturers, regulators, and implementers. This combination of perspectives from the scientific, commercial and public health worlds can support more informed decision making [34].

In order to reach multiple countries, the involvement of WHO headquarters, country and, in particular, regional offices has been and will remain critical, particularly for diseases such as HIV/AIDS, TB and malaria that have a significant number of WHO staff. Regional WHO offices can track the progress of multiple countries as they move through the multi-part decision process. Table 4 describes possible partners, including WHO, and some of their advantages and disadvantages.

**Table 4:** Partners who can support country decision making, in collaboration with PDPs

Partner	Advantages	Disadvantages
Multilaterals such as WHO	Extensive reach and impartiality	Limited staff and restricted funding; May be overwhelmed by other initiatives and thus lack time and resources to devote to new interventions

Organizations dedicated to new product access, such as those funded by GAVI (e.g., Hib Initiative, Accelerated Vaccine Introduction Initiative (AVI))	Dedicated funding for access activities	Typically have a multi-country remit which limits depth of engagement in individual countries

Local academia, researchers and/or professional organizations	Close to in-country processes, needs and data; Credible with local policy-makers	May not have a broad view of a problem; May be influenced by personal research interests

NGOs	Some have specific expertise in new product introduction	May require funding specific to the new product to support their activities, and may not be involved in official decision-making bodies

Pharmaceutical and/or manufacturing partners	Product-specific expertise, regulatory expertise, and in some cases extensive sales networks in some markets	May be seen as a biased source of decision making information; may lack experience in the disease and/or in low and middle income settings

Manufacturers have traditionally supported some aspects of country decision-making and all the aspects of product launch. However, many originator companies may have limited experience of introduction into low and middle income country markets (some Indian and Chinese generics may be more established in these markets). Companies may also be concerned that they could be perceived to be self-serving if supporting decision-making around the introduction of a new product directly in countries. Thus, the initial information sharing and country decision making step will generally require the involvement of other actors, including PDPs.

An interesting example of division of labor comes from Uganda, where PATH, a not-for-profit that has worked extensively as a PDP, is supporting a demonstration project for human papilloma virus (HPV) vaccines (E. Mugisha, pers. comm.). Before any activities started, PATH signed a Memorandum of Understanding (MOU) with the Government of Uganda (GoU) to specify who would do what. The GoU committed to provide health services delivery infrastructure, human resources in the districts, and EPI staff for delivery of the vaccine. The two PATH technical staff members, located in the WHO Uganda office, provided technical and logistical support. PATH also provided transport allowances (but no per diems) to health workers in the field and funded local university researchers to conduct the formative research and operations research. WHO and UNICEF participated in a technical advisory committee set up by the Ministry of Health (MoH) to oversee the demonstration project, and also helped with monitoring of vaccination. The relevant pharmaceutical company (GlaxoSmithKline Biologicals (GSK)) donated and shipped vaccines to Uganda, but had no other role in the project. UNFPA and other stakeholders provided input on reproductive health issues, and NGOs (e.g., CARE and Save the Children) helped with mobilization in the districts. Thus, PATH served as the glue across the various organizations in support of the MoH.

## Challenges to PDP implementation

There are many challenges for PDPs in supporting country decision making. Creating a success story in one location is certainly important, but just getting the process right in one country won't necessarily allow replication, as each country is different. The PDP, however, is unlikely to have the resources to replicate the same breadth of activities with all endemic countries.

There is also an issue of managing expectations. Country decision making is driven by the country, not the PDP. Conveying this idea of public sector policy change to R&D staff, PDP boards and funders can be challenging. Furthermore, PDP boards often think that local implementation partners can do it all, so local engagement by the PDP is not necessary. But partners are focused on many other issues, and often do not have the full depth of information on a given intervention.

Finally, optimal engagement timelines are unclear. Advance planning is risky as product timelines are uncertain, but without early engagement (e.g. 5 years pre-licensure), country decision making may be delayed and products will sit unused on shelves. In terms of PDP access budgets, a critical step will be to address the number and cost of Phase 4 studies, determine who will bear the burden of financing them (e.g., donors and PDPs, manufacturers, countries or some combination), and define models to bring those costs down.

## Specific PDP approaches for supporting country decision making activities

Despite these challenges, successful PDP support of country decision making is possible. The case studies below illustrate that PDPs have taken many distinct approaches to facilitating country decision making. These are examples of what has been done, rather than normative descriptions of what would be ideal. They were selected to represent a range of modalities (e.g., vaccine, drug, and insecticide) and disease areas, and the kinds of activities conducted at different stages of the decision process (presented below in roughly chronological order). Different PDPs have undertaken a range of activities, such as those shown in Tables 1 and 2, with this selection depending on needs identified by each PDP, typically in consultation with partners like WHO and countries. Not surprisingly, the more extensive experience generally lies with the PDPs who have approved products.

### Engagement prior to product availability

For DNDi, country engagement begins with the identification of needs by and with endemic country stakeholders. Certain key research organizations in endemic countries contributed to DNDi's founding, are represented on its Board of Directors, and greatly inform the definition of needs and of the related Target Product Profiles (TPPs). These organizations also facilitate clinical and intervention trials and, ultimately, national decisions on adoption.

Three additional PDPs, whose activities are outlined below, do not yet have products approved by regulatory agencies but nonetheless conduct activities related to country decision making. For malaria vaccines, MVI initiated a 3 year process with countries, WHO and, in the later stages, the Roll Back Malaria Partnership (RBM) to develop decision-making frameworks, initially for 9 individual countries and then for the African region [33]. The final framework builds upon existing WHO guidelines [22], and lays out what data are needed from different sources (global vs national), in different thematic areas (disease burden, other malaria interventions, impact, financial, efficacy, safety, programmatic, sociocultural), and at different times (pre-licensure, licensure, and post-licensure); it also notes whether each is essential or desirable. It provides a similar framework for policy processes.

The framework process has and will structure dialogues around malaria vaccines with countries around technical issues. In some countries, it has led to the formation of ongoing structures that have begun to collect data to inform an eventual decision (e.g., technical working groups - MVI now sponsors three of these).

In contrast to MVI, the Global Alliance for TB Drug Development (TB Alliance) is entering an area that has existing products. There was, however, relatively little analysis of the market, of decision making, or of how new products would be considered. TB Alliance therefore focused on conducting sequential stakeholder studies in the following areas: the size and structure of the existing TB drug market; what local stakeholders want from a new TB regimen; how the experience with past TB regimen changes can inform future approaches; and what producers and products are dominant in the private sector. These studies helped to identify issues and categories of data relevant for future country decision making [12,31], initiate engagement with local stakeholders, provide opportunities for the promotion of regimen change issues in international fora, and frame conversations with local stakeholders during TB Alliance participation in WHO review missions. Finally, the findings of each study influenced the design and content of the next, and provided essential feedback for the research and development team [2]. TB Alliance selected this approach due to the opportunities and challenges presented by the availability of existing TB treatment regimens and partnerships.

The long-term aim of IVCC is to facilitate the development and introduction of new insecticides. Already, however, IVCC is engaging country decision makers to address an identified gap in field implementation - the monitoring and evaluation of vector control programs. This gap is addressed via IVCC's Malaria Decision Support System (MDSS), which is used to track clinical and survey data and insecticide resistance (T. McLean, pers. comm.). This tool is applicable to a wide range of diseases and, in addition to monitoring and evaluation, it supports the management of advanced insecticides, and decision making on adopting new vector control products. Based on existing partnerships with the ministries of health, IVCC has validated the methodology in 3 countries - Mozambique, Malawi, and Zambia - with varied infrastructure and ecological environments; it is now planning wide-ranging implementation. As a central objective, the MDSS should be adopted and owned by the national malaria control program and serve their information needs.

### Country consultations and regional meetings

Early and frequent consultations with countries are essential for the development of products that are suited to end users [2]. For DNDi, input is channeled via disease platforms, which were formed to assist and strengthen clinical research around specific diseases in a geographic area, e.g., Visceral Leishmaniasis (VL) in East Africa, sleeping sickness in West Africa, and Chagas Disease in Latin America. These platforms include country program staff, researchers, regulatory officials, NGOs and WHO and meet twice a year. Platform members became natural partners for country decision making as they gather relevant information on in-country issues, programs, and processes and convey key information to in-country decision makers.

The Pneumo-ADIP (now part of the International Vaccine Access Center (IVAC)) built on experience with Hib and Hepatitis B vaccine introductions to support pneumococcal vaccine introduction [9]. Under the Pneumo-ADIP, the establishment of surveillance networks had two positive outcomes: it provided the requested data on projected coverage and impact and, via annual investigators' meetings, led to the identification of local advocates. In addition, regional meetings organized in collaboration with WHO provided an opportunity to check back in and to move countries to put their decisions and proposed actions on paper by presenting their conclusions in front of others (L. Privor-Dumm, pers. comm.). These meetings included EPI managers, directors of health services, researchers, pediatricians, economists, and sometimes donors and financing people from MoH or other ministries, and were a particularly useful mechanism to support decision-making in countries not directly targeted through other interactions.

MMV and its drug development partners have also made extensive use of country-level dialogues such as subregional meetings (of WHO AFRO and Roll Back Malaria) and, in select cases, day-long workshops (G. Jagoe, pers. comm.). These provide opportunities to give product-specific briefings and to reinforce recommendations of normative entities (primarily WHO) in terms of best practice for the development and revision of treatment guidelines and for the correct use of new, quality medications in combination with proper diagnosis (case management). Longer-term programmatic collaborations in specific-countries are very limited; the focus is on any initiative (e.g., piloting of an affordable medicines private sector subsidy in Uganda) that address specific access challenges and could serve as guiding lights for policy makers and funders across the larger stage of all malaria endemic countries. In terms of impact, as of September 2010, almost 42 million treatments of Coartem^® ^Dispersible (co-developed with MMV) had been delivered to 32 countries [37].

Regional meetings have been convened by the TB Alliance and partners to gain consensus around regulatory issues [38]. Existing TB drugs were developed more than 40 years ago, in a very different regulatory environment. Agreement was needed on the regulatory approach to development of not just individual new drugs, but new regimens. With the participation of national TB program managers in these meetings, these individuals became part of the conversation about what types of evidence would be available for decision making.

### Implementation studies as a bridge to adoption

When existing evidence is insufficient, implementation studies may be necessary. In India, the Institute for One World Health (iOWH) has supported studies to generate data for advocacy and decision making on VL treatment and elimination. In collaboration with a research institute of the Government of India, they documented the incidence of VL, the financial burden of disease, households' willingness and ability to pay, and treatment-seeking behaviors in both public and private sectors (R. Sarnoff, pers. comm.). In addition, building on the necessary Phase 3 study, iOWH sponsored a Phase 4 study with an effectiveness module that provided training, clinical support, and guidance on pharmacovigilance reporting, and demonstrated effective delivery in public and private facilities. The clinical trial investigators formed a core constituency for local advocacy for improved products.

At the national level, iOWH leadership engaged with key stakeholders in the Indian government, World Bank and WHO to inform them of the progress of the studies, identify their key questions and concerns, and address future funding issues. Training modules and community communication models were developed for smooth transfer to the national authorities.

DNDi has also used intervention or field trials as an essential step to demonstrate feasibility and generate necessary data for adoption into national programs (F. Camus-Bablon, pers. comm.). For example, Brazil conducted a 25,000-subject malaria intervention trial prior to adopting artesunate and mefloquine (ASMQ) for treatment of falciparum malaria in the Amazon basin. The trial monitored the effects of ASMQ introduction; during the study, a significant impact on malaria cases and related hospitalization also resulted from a more rational use of complementary resources such as insecticides, a detection and reporting system, and the training of local human resources. In this study, one year after the introduction of the ASMQ fixed-dose combination (FDC) and the treatment of 17,000 patients, *P. falciparum *malaria cases were reduced by nearly 70% and malaria-related hospitalizations dropped by over 60%. Following the study, the Brazilian National Program updated the national malaria treatment guidelines and introduced ASMQ FDC as the first line treatment in the region. Advocacy is also a key component of DNDi implementation work, to inform both international audiences and endemic countries.

In some areas, DNDi relies on pharmaceutical and other international partners. For example, WHO Neglected Tropical Diseases (NTD) department and Médecins Sans Frontières (MSF) are key drivers for the adoption of nifurtimox-eflornithine combination therapy (NECT) for the treatment of sleeping sickness, which, within a year, has been adopted in the national treatment policy of nine endemic countries and ordered by six. Sanofi-Aventis (SA), within two years of WHO pre-qualification, was planning to distribute 50 million artesunate-amodiaquine (ASAQ) treatments in 2010. Today, ASAQ is registered in 27 African countries and in India. SA is conducting a 15,000 patient pharmacovigilance program in partnership with DNDi and MMV in Ivory Coast, and developed a specific package for social interventions and home based management programs.

### Evaluation of impact

There are significant challenges in estimating the impact of PDPs on country decision making. First, most PDPs are relatively young, being established in the last 10 years. Given the time required to develop a product, many have not yet had products launched, and the product launches that have occurred are recent. Second, there is not yet agreement on how to measure impact - in particular, whether to focus on usage (e.g., number of individuals treated; see the data on new product usage noted in this paper) or on process (the number of countries conducting a policy process and reaching the decision that is best for their particular situation; see [39] for an example). Third, if a product is suboptimal - too expensive, insufficiently efficacious, or too difficult to use, for example - it is unlikely to be rescued by PDP support of country decision making, even if those support activities are well executed. In other words, success under the first ("usage") definition is only likely when the new product continues to meet identified country needs. This is why access input is essential throughout the product development process [2].

There have, however, been efforts by PDPs to define metrics of success and to evaluate PDP work in support of country decision-making. For impact using the "process" definition of success, one example is the malaria vaccine decision-making framework [39]. The development of this framework has been independently evaluated [40]. Out of 84 respondents from 10 countries, 90% felt that the framework developed will be extremely or very useful in preparation for a decision (i.e., in deciding what activities to undertake prior to having a licensed product), and 88% indicated the same for taking a decision after a vaccine is licensed. Facilitators were reported to be neutral instead of supporting one product.

A 2007 study by the GAVI Alliance tried to quantify the impact of the Pneumo- and Rotavirus ADIPs and Hib Initiative as compared to what may have happened if they had not been in place [41]. The authors found that the Pneumo-ADIP is likely to shave at least five years off the time from development to availability of vaccines in the poorest countries, and that the work of the Rotavirus ADIP may result in the poorest countries accessing vaccines only one year after availability in the developed world, which is years and decades shorter than historically. Based upon this, the authors reported "value in terms of lives saved and hospitalizations averted."

Given the constraints noted above, the current paper does not aim to provide a rigorous evaluation of PDP impact and strategies, but instead provides a situation analysis, reflecting the range of strategies undertaken by PDPs and detailing the rationale behind their choice. It is clear that having no engagement specifically around new products leads to lengthy delays in availability [1]. At the same time, it is too early to determine the optimal strategies for each situation and type of intervention. However, the current analysis provides an important baseline or reference point for a later impact analysis of PDP work on access.

## Conclusion

A country's decision to adopt a new health technology requires more than the existence of a good product. In low and middle income settings, a wide range of organizations can support country decision making. The role of PDPs in this process is based on the PDPs' vision to see public health impact from the products they develop, and on their intimate familiarity with the products under discussion.

A PDP as a whole can cover a wide spectrum of activities ranging from basic research to implementation of interventions. At the implementation end of this spectrum, there is no single definition of where the PDP role ends, as the technical needs and available partners in endemic countries vary for each intervention. However, as more PDP-related products progress, additional experience will assist in defining the areas in which PDPs are effective and should be held accountable. Funder, partner and country participation in the development of improved means to evaluate the relative roles and impact of PDPs will also be important. Building on the insights described here, PDPs, partners and country stakeholders can continue to provide critical support for decisions on interventions that will ultimately decrease the global burden of disease.

## Competing interests

The authors are or have been employed by product development partnerships. The authors declare that they have no other competing interests.

## Authors' contributions

WAW initiated the review concept, analyzed suggested inputs from various organizations, wrote the first draft of the manuscript, and revised subsequent drafts; AB helped analyze the suggested inputs, contributed key concepts, and revised manuscript drafts. Both authors read and approved the final manuscript.

## Authors' information

WW is Director, Market Access, at the Global Alliance for TB Drug Development (TB Alliance); AB was formerly Director, Policy and Access, PATH Malaria Vaccine Initiative and is currently a doctoral student at the Swiss Tropical and Public Health Institute.
